# Tet1-dependent epigenetic modification of BDNF expression in dorsal horn neurons mediates neuropathic pain in rats

**DOI:** 10.1038/srep37411

**Published:** 2016-11-18

**Authors:** Ming-Chun Hsieh, Cheng-Yuan Lai, Yu-Cheng Ho, Hsueh-Hsiao Wang, Jen-Kun Cheng, Yat-Pang Chau, Hsien-Yu Peng

**Affiliations:** 1 Department of Physiology, College of Medicine, National Taiwan University, Taipei, Taiwan; 2 Department of Medicine, Mackay Medical College, New Taipei, Taiwan; 3 Department of Veterinary Medicine, College of Veterinary Medicine, National Chung-Hsing University, Taichung, Taiwan; 4 Department of Anesthesiology, Mackay Memorial Hospital, Taipei, Taiwan

## Abstract

Ten-eleven translocation methylcytosine dioxygenase 1 (Tet1) mediates the conversion of 5-methylcytosine (5 mC) to 5-hydroxymethylcytosine (5 hmC), hence promoting DNA demethylation. Although recent studies have linked the DNA demethylation of specific genes to pain hypersensitivity, the role of spinal Tet1-dependent DNA demethylation in nociception hypersensitivity development remains elusive. Here, we report correlated with behavioral allodynia, spinal nerve ligation (SNL) upregulated Tet1 expression in dorsal horn neurons that hydroxylate 5 mC to 5 hmC at CpG dinucleotides in the *bdnf* promoter to promote spinal BDNF expression at day 7 after operation. Focal knockdown of spinal Tet1 expression decreased Tet1 binding and 5 hmC enrichment, further increased 5 mC enrichment at CpG sites in the *bdnf* promoter and decreased spinal BDNF expression accompanied by the alleviation of the developed allodynia. Moreover, at day 7 after operation, SNL-enhanced Tet1 expression also inhibited the binding of DNA methyltransferases (DNMTs, i.e., DNMT1, DNMT3a, and DNMT3b) to the *bdnf* promoter, a requirement for transcriptional silencing by catalysing 5-cytosine (5C) to 5 mC. Together, these data suggest at CpG sites of the *bdnf* promoter, SNL-enhanced Tet1 expression promotes DNA demethylation both by converting 5 mC to 5 hmC and inhibiting DNMT binding to regulate spinal BDNF expression, hence contributing to behavioral allodynia development.

Neural plasticity in the spinal nociceptive pathway relies on molecular machineries similar to learning/memory-associated plasticity in brain areas to cause pain hypersensitivity and is mediated, at least partially, by an epigenetic process^1^. Although a study has linked DNA demethylation epigenetic modification to painful diabetic neuropathy^2^, the detailed machinery concerning how active DNA demethylation impacts the spinal plasticity underlying neuropathic pain development remains unclear. The ten-eleven translocation methylcytosine dioxygenase family (Tet1-3), which possesses 2-oxoglutarate and Fe(II)-dependent oxygenase activities, has been identified to convert 5-methylcytosine (5 mC) to 5-hydroxymethylcytosine (5 hmC) in the CpG dinucleotide, and thereby initiate active DNA demethylation epigenetic process^3^,^4^,^5^. Among the Tet families, Tet1 is recognized to be essential for the development of neural plasticity because Tet1-dependent DNA demethylation reactivates methylation-silenced genes at plasticity-associated genomic loci^5^,^6^,^7^. Viral-mediated Tet1 overexpression that increases the conversion of 5 mC to 5 hmC results in the upregulation of memory-associated genes in neurons^5^. Conversely, Tet1-knockout mice present learning deficiencies and memory retention accompanied by extensive methylation of cytosine in the promoters of learning/memory-related genes^8^. Recently, a study reported that spinal Tet family are essential for inflammatory nociception transmission^9^. These evidence implying that analogous forms of synaptic plasticity caused by the Tet1-mediated DNA demethylation-modifying 5 mC/5 hmC enrichment in pain-associated genes may operate at spinal sites to participate in neuropathic pain processing.

Increasing evidence has demonstrated that neuronal activity-induced Tet1 expression is necessary for the demethylation of genes that mediates neural functions such as the brain-derived neurotrophic factor (*bdnf*) gene^6^. For example, Tet1 overexpression leads to increased 5 hmC but decreased 5 mC enrichment at CpG sites in the *bdnf* promoter; conversely, Tet1 downregulation is associated with reduced levels of 5 hmC enrichment at CpG sites in the *bdnf* promoter to decrease BDNF expression^10^. Numerous studies have linked *bdnf* transcription to the spinal plasticity underlying neuropathic pain in rat models^11^,^12^. Altogether, these observations imply that Tet1-mediated DNA demethylation at CpG sites in the *bdnf* promoter could underlie the spinal plasticity causing the neuropathic pain.

In addition to Tet1-dependent demethylation, which converts 5 mC to 5 hmC, Levenson and colleagues^13^ demonstrated that the DNA methylation status at the *bdnf* promoter is also regulated by DNA methyltransferases (DNMTs), well-recognized methylation regulators that promote 5-cytosine methylation in DNA. Recent studies have demonstrated that Tet1 regulates demethylation both by converting 5 mC to 5 hmC and by inhibiting DNMT binding to CpG sites in DNA; the Tet1-generated 5 hmC also inhibits DNMT binding to DNA, thus allowing a 5 hmC-marked region to escape from heterochromatinization^14^. Conversely, Tet1-knonckout mice manifest increased DNMT expression in memory-associated brain areas^15^. Among DNMT family members, DNMT1 maintains gene methylation, DNMT3a and DNMT3b mediate de novo gene methylation, and all of them are preferentially expressed in neurons to pivotally contribute to pain hypersensitivity^16^,^17^. Thus, spinal Tet1-achieved demethylation likely represents one mechanism to negatively modulate DNMTs-mediated methylation at the *bdnf* promoter to mediate neuropathic pain development. Here, we investigated the contribution of spinal Tet1 to the epigenetic machinery underlying neuropathic pain and the possible involvement of Tet1-promoted 5 mC/5 hmC conversion-associated gene modification.

## Results

### SNL enhances ipsilateral spinal Tet1 expression

**Figure 1:**
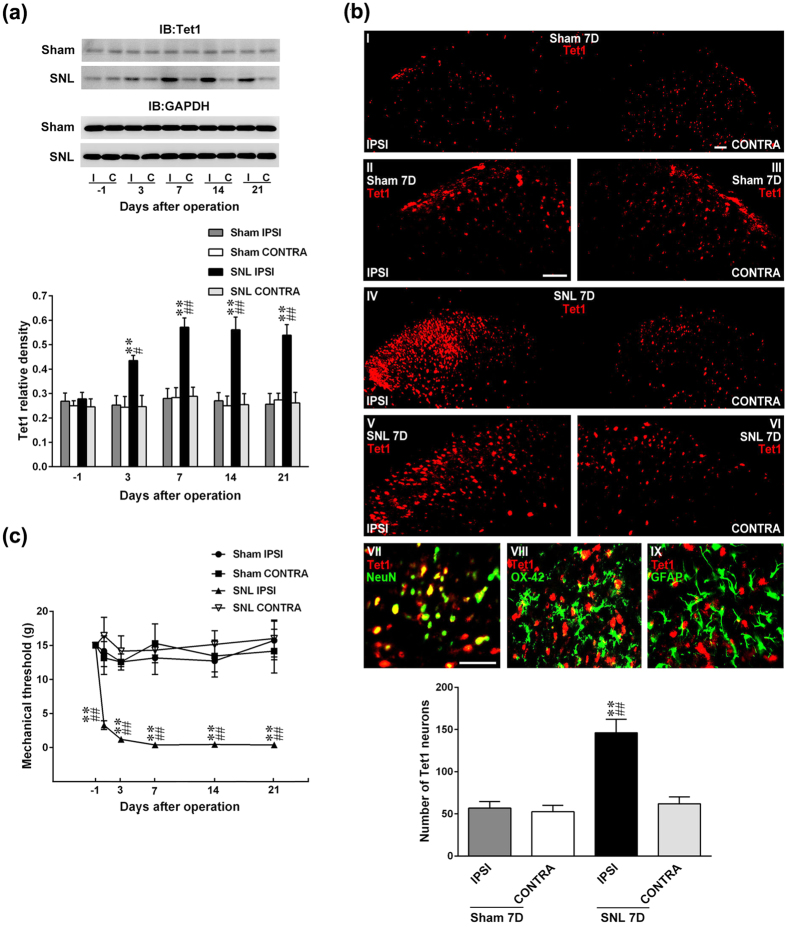
SNL enhances Tet1 expression in ipsilateral dorsal horn neurons accompanied by behavioral allodynia. (**a**) Representative Western blot and statistical analyses (normalized to GAPDH) revealing that spinal nerve ligation (SNL), but not the sham operation (Sham), increased Tet1 abundances at days 3, 7, 14, and 21 after operation specifically in the ipsilateral (I and IPSI), but not the contralateral (C and CONTRA), dorsal horn. **P < 0.01 vs. Sham IPSI. ^#^P < 0.05, ^##^P < 0.01 vs. SNL day -1. n = 6. IB, Immunoblotting. (**b**) Representative images showing the SNL-increased Tet1 immunofluorescence (red) in the ipsilateral dorsal horn at day 7 post-operation (SNL 7D) that was colocalized with the immunoreactivity of the neuronal (NeuN, green, VII) marker, but not with that of the (OX-42, green, VIII) or astrocyte (GFAP, green, IX), marker. All of the scale bars = 50 μm. Thickness = 50 μm. **P < 0.01 vs. Sham IPSI, ^##^P < 0.01 vs. SNL CONTRA. n = 7. (**c**) The withdrawal threshold of the ipsilateral, but not the contralateral, hindpaw was decreased at days 1, 3, 7, 14, and 21 after SNL (von Frey test). **P < 0.01 vs. Sham IPSI. ^##^P < 0.01 vs. SNL day -1. n = 7.

To characterize the role of spinal Tet1 in neuropathic pain, we initially analysed Tet1 expression in dorsal horn samples dissected from rats with spinal nerve ligation (SNL) and sham operation (Sham). In contrast to a relatively uniform level of Tet1 in the contralateral side, SNL increased the Tet1 expression in the ipsilateral dorsal horn. The enhanced Tet1 expression onset at day 3, became maximal at day 7, and then maintained until days 14 and 21 after nerve ligation (Fig. 1a). In addition, at day 7 after SNL, the upregulated Tet1 expression in the ipsilateral dorsal horn was also identified by immunohistochemistry analysis (Fig. 1b). Double-labelled images revealed the Tet1 immunoreactivity in the dorsal horn coincides with that of neuronal but not microglial or astrocytic markers. Moreover, in the ipsilateral hind paw, SNL reduced the withdrawal threshold at days 3, 7, 14, and 21 post-operation that was consistent with the time profile of the SNL-enhanced Tet1 expression, (Fig. 1c). These data suggested the neuropathic injury provokes behavioral allodynia in accompanied with ipsilateral dorsal horn Tet1 expression.

### Spinal Tet1 knockdown alleviates neuropathic allodynia

**Figure 2:**
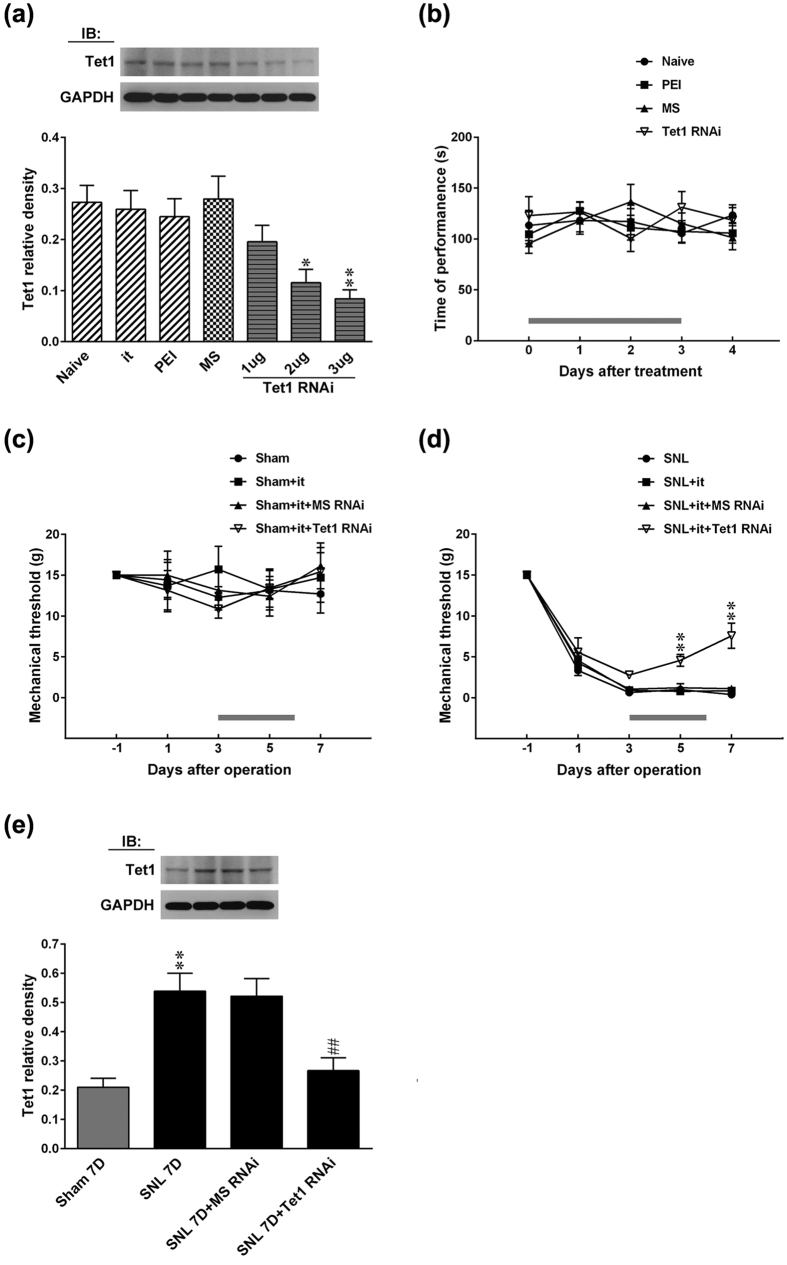
Focal knockdown of spinal Tet1 relieves SNL-induced allodynia without affecting motor function. (**a**) Representative Western blot and statistical analyses (normalized to GAPDH) demonstrating the intrathecal administration of Tet1 mRNA-targeting siRNA (Tet1 RNAi; 1, 2, and 3 μg; 10 μL; once daily for 4 days), but not missense siRNA (MS, 3 μg, 10 μL), polyethylenimine (PEI, 10 μL) or intrathecal catheter implantation alone (it), dose-dependently decreased spinal Tet1 expression in naïve rats (Naïve). *P < 0.05, **P < 0.01 vs. Naive. n = 6. IB, Immunoblotting. (**b**) The application of Tet1 mRNA-targeting siRNA (3 μg, 10 μL) resulted in no motor deficits in animals (rotarod test). The grey bar at the bottom indicates the duration of administration. n = 7. (**c**,**d**) Tet1 mRNA-targeting siRNA (SNL + it + Tet1 RNAi, 3 μg, 10 μL; once daily from day 3–6 after operation) reversed the SNL-induced allodynia at days 5 and 7 post-operation, but did not affect the withdrawal threshold of the sham-operated animals (Sham + it + Tet1 RNAi, 3 μg, 10 μL) (von Frey test). The grey bar at the bottom indicates the duration of administration. **P < 0.01 vs. SNL. n = 7. (**e**) Representative Western blot and statistical analyses (normalized to GAPDH) showing that, at day 7 post-operation, the SNL-enhanced spinal Tet1 expression was attenuated by Tet1 mRNA-targeting siRNA (SNL + it + Tet1 RNAi, 3 μg, 10 μL; once daily from day 3–6 after operation). **P < 0.01 vs. Sham 7D. ^##^P < 0.01 vs. SNL 7D. n = 6.

We next tested if genetic trim-down of dorsal horn Tet1 expression impacts SNL-induced allodynia to confirm the role of spinal Tet1 in neuropathic pain development. In the ipsilateral dorsal horn of naive rats, Western blotting analysis revealed the Tet1 expression was dose-dependently (1, 2, and 3 μg; 10 μL; once daily for 4 days) decreased by spinal application of Tet1 mRNA-targeting siRNA (Fig. 2a), but not by the application of missense siRNA (3 μg; 10 μL), the application of polyethylenimine (the transfection reagent; 10 μL), or the implantation of intrathecal catheter. This result revealed that our antisense siRNA efficiently trimed-down spinal Tet1 expression. Results of rotarod test demonstrated that there was no significant difference between groups (Fig. 2b). These results indicate that neither the experimental procedure nor the administration of antisense siRNA resulted in motor deficits. While daily application of Tet1 mRNA-targeting siRNA (3 μg; 10 μL; days 3–6 post-SNL) did not affect the withdrawal threshold of sham-operated animals (Fig. 2c), it partially reversed SNL-associated allodynia, as evidenced by significantly increasing the withdrawal threshold at days 5 and 7 after SNL (Fig. 2d). Moreover, administration with Tet1 mRNA-targeting siRNA significantly reversed the SNL-enhanced Tet1 expression in the ipsilateral dorsal horn at day 7 (Fig. 2e).

### Spinal Tet1 knockdown alleviates SNL-enhanced dorsal horn neuron excitability

**Figure 3:**
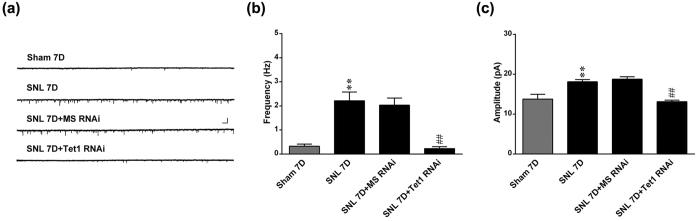
Focal knockdown of spinal Tet1 alleviates SNL-enhanced dorsal horn neuron excitability. (**a**–**c**) EIectrophysiological recording traces and statistical analyses shown that compared with the sham operation, SNL increased the frequency and amplitude of mEPSCs of dorsal horn neurons that were reversed by daily administration with Tet1 mRNA-targeting, but not antisense siRNA. Scale bar = 30 pA, 300 ms. **P < 0.01 vs. Sham 7D. ^##^P < 0.01 vs. SNL 7D. n = 5.

When compared with the sham operation, SNL significantly increased the frequency and amplitude of mEPSCs recorded from the dorsal horn neurons of spinal slices that were both reversed by daily administration with Tet1 mRNA-targeting siRNA (3 μg; 10 μL; day 3–6 post-SNL) but not the missense siRNA (Fig. 3a,b,c). These results indicate that neuropathic injury enhanced excitability of dorsal horn neuron through spinal Tet1-dependent pre- and post-synaptic machineries.

### SNL enhances the global 5 hmC and 5 mC levels in the ipsilateral dorsal horn

**Figure 4:**
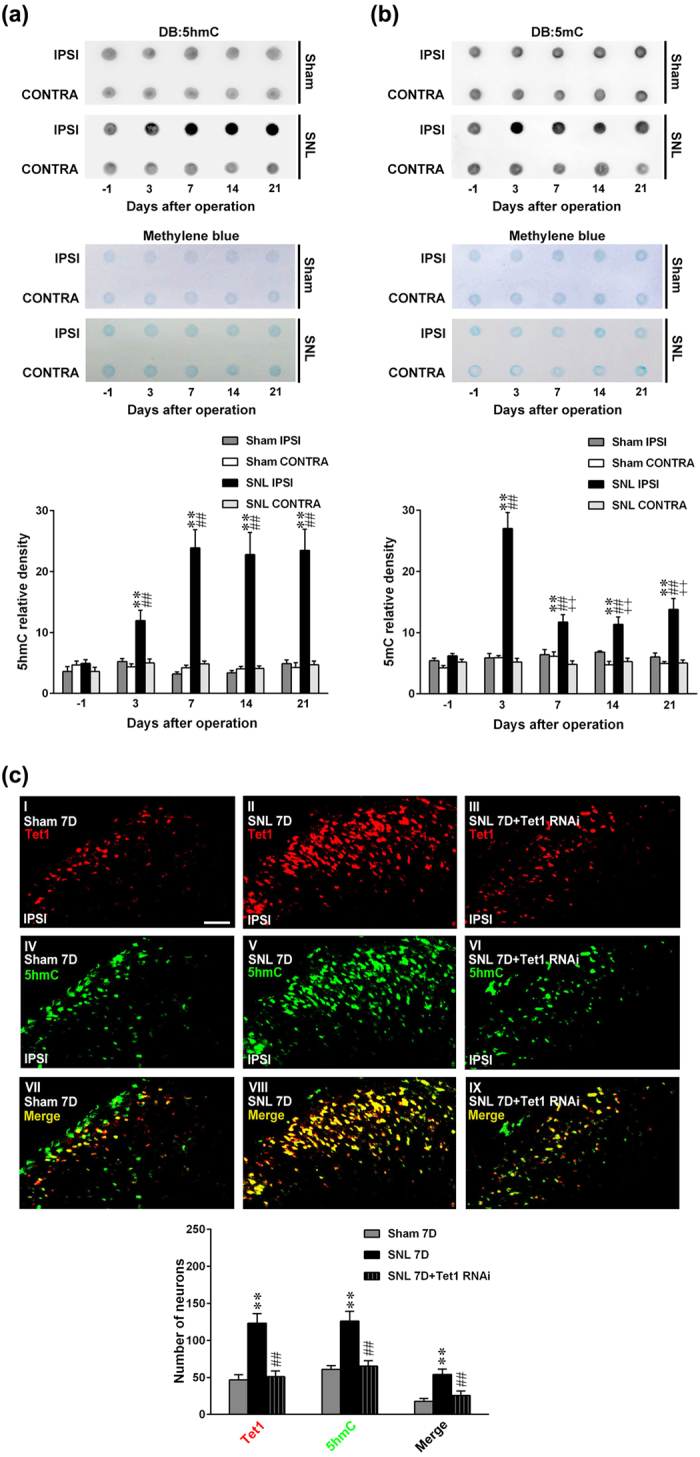
SNL enhances the global amounts of 5 hmC and 5 mC in the ipsilateral dorsal horn. (**a**,**b**) The top panel shows the representative dot blot analyses of the global amounts of 5 hmC and 5 mC from the ipsilateral (IPSI) and contralateral (CONTRA) dorsal horn of spinal nerve ligation (SNL) and sham-operation (Sham) rats, respectively. The middle panel is methylene blue staining to validate equal loading of DNA. The bottom panel shows statistical analysis of the relative intensity of 5 hmC and 5 mC in dot blot analyses in both groups. DB, Dot blotting. **P < 0.01 vs. Sham IPSI. ^##^P < 0.01 vs. SNL day -1. n = 6. ^++^P < 0.01 vs. SNL day 3. n = 6. (**c**) Image analyses demonstrate that, compared with the sham operation (Sham 7D), SNL (SNL 7D) markedly increased the Tet1 (red), 5 hmC (green), and Tet1/5-hmc double-labelled (yellow) immunofluorescence in the ipsilateral dorsal horn at day 7 post operation, activities that were all reversed by the daily administration of Tet1 mRNA-specific antisense siRNA (Tet1 RNAi; 3 μg, 10 μL, from day 3 to 6 after operation). **P < 0.01 vs. Sham 7D. ^##^P < 0.01 vs. SNL 7D. n = 7. Scale bar = 50 μm. Thickness = 50 μm.

Emerging evidence has linked DNA demethylation to the development of chronic pain^3^,^18^, and a recent study showed that Tet1 promotes DNA demethylation by producing global 5 hmC in hippocampal neurons^5^. Based on the above results revealing that SNL-enhanced spinal Tet1 expression crucially contributes to neuropathic pain, we next assayed the global level of spinal 5 hmC in response to neuropathic injury. As anticipated, the global level of 5 hmC in the ipsilateral dorsal horn increased in proportion to the SNL-enhanced Tet1 expression—i.e., it gradually increased at day 3, reaching its peak at day 7, and then maintaining this level at days 14 and 21 after operation (Fig. 4a), suggesting that SNL-enhanced spinal Tet1 expression may promote DNA demethylation by producing global 5 hmC. On the other hand, Tet1 is recognized to hydroxylate 5 mC into 5 hmC to initiate a DNA demethylation epigenetic process^4^; thus, we next examined the impact of neuropathic injury on the amount of global 5 mC. Interestingly, although SNL enhanced global 5 mC in the ipsilateral dorsal horn in the progression of neuropathic pain (Fig. 4b), we found that the expression of SNL-enhanced global 5 mC was initiated and reached a peak at day 3 but was significantly blunted at days 7, 14, and 21 compared with that at day 3 after SNL. Additionally, in the ipsilateral dorsal horn of slice obtained at day 7 post-operation, immunofluorescence images revealed that daily application of Tet1 mRNA-specific antisense siRNA (3 μg, 10 μL) to SNL animals prevented Tet1 and 5 hmC upregulation, and the colocalized immunoreactivity (Fig. 4c). Collectively, we speculate that the enhanced global 5 hmC could be attributed to the Tet1-dependent hydroxylation of the SNL-increased spinal global 5 mC level.

### SNL enhances Tet1-mediated 5 mC/5 hmC conversion at the CpG sites of the *bdnf* promoter in the ipsilateral dorsal horn

**Figure 5:**
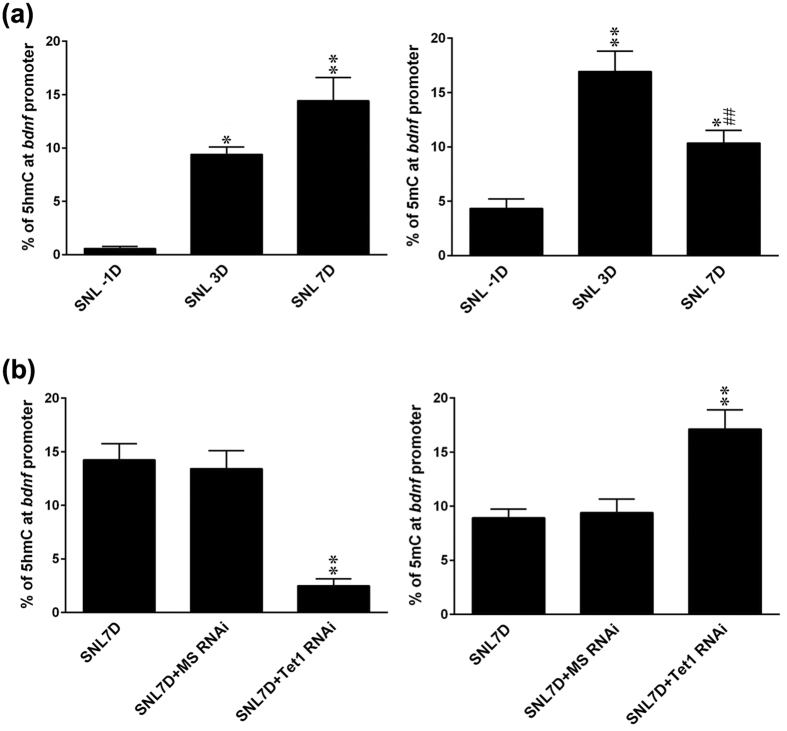
SNL induces Tet1-modified 5 mC and 5 hmC enrichment at the CpG sites of the *bdnf* promoter in the ipsilateral dorsal horn samples. (**a**) Representative statistical analyses revealing that SNL-increased 5 hmC (left) and 5 mC (right) enrichment at the *bdnf promoter* at days 3 and 7 post-operation. Notably, while the 5 hmC enrichment was higher at day 7 than day 3, SNL-increased 5 mC enrichment was significantly decreased at day 7 compare with that at day 3 after SNL. *P < 0.05, **P < 0.01 vs. SNL -1D. ^##^P < 0.01 vs. SNL 3D. n = 10. (**b**) While it significantly decreased the 5 hmC enrichment, the administration of Tet1 mRNA-targeting siRNA (SNL + it + Tet1 RNAi, 3 μg, 10 μL; from day 3–6 after SNL) to SNL animals further increased the 5 mC enrichment at the CpG sites of the *bdnf promoter* at day 7 post-SNL. **P < 0.01 vs. SNL 7D. n = 10.

Emerging evidence has suggested that Tet1 and 5 hmC are particularly enriched at the transcription start sites with high CpG content^19^. Notably, Tet1 overexpression promotes 5 mC/5 hmC conversion at the CpG sites in the brain-derived neurotrophic factor (*bdnf*) promoter^10^. Considering that BDNF-dependent neural plasticity in the spinal cord has been linked to the genesis of persistent pain^11^,^12^, we wondered whether spinal Tet1-modified 5 mC and/or 5 hmC enrichment at the CpG sites in the *bdnf* promoter mediated SNL-induced neuropathic pain. In line with the above global data, we found that 5 hmC enrichment at the CpG sites in the *bdnf* promoter in the ipsilateral dorsal horn was time dependently increased at day 3 and day 7 post SNL (Fig. 5a), time points presumed to reflect the induction^20^ and maintenance^21^ phases of neuropathic pain, respectively. On the other hand, although 5 mC enrichment was also increased at days 3 and 7 it was significantly blunted at day 7 compared with that at day 3 after SNL, suggesting that SNL may promote the hydroxylation of 5 mC to 5 hmC at the CpG sites in the *bdnf* promoter during the maintenance phases of neuropathic pain. To further assess whether Tet1 catalyse 5 mC hydroxylation and, hence, attenuate SNL-enhanced 5 mC enrichment via active DNA demethylation, we daily administered SNL rats with Tet1 mRNA-targeting siRNA through the intrathecal catheter. Interestingly, in the ipsilateral dorsal horn sample obtained at day 7 post-surgery, focal knockdown of spinal Tet1 expression (3 μg; 10 μL; once daily from day 3–6 post-SNL) significantly decreased 5 hmC but increased 5 mC enrichment at the CpG sites in the *bdnf* promoter (Fig. 5b), revealing the presence of spinal Tet1-dependent 5 mC conversion to 5 hmC at the CpG sites in the *bdnf* promoter during the maintenance phases of neuropathic pain.

### SNL promotes spinal Tet1-dependent *bdnf* transcription

**Figure 6:**
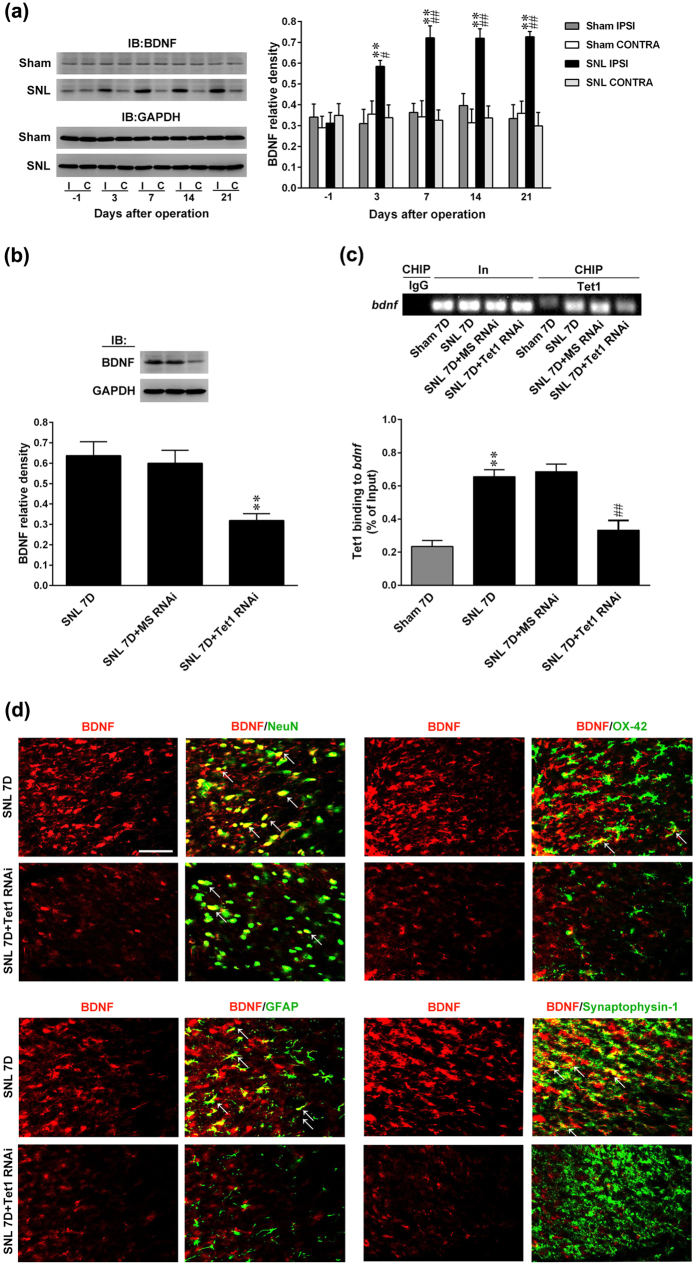
SNL induction of spinal BDNF expression and Tet1-*bdnf* promoter binding were reversed by focal knockdown of spinal Tet1 expression. (**a**) Representative Western blot and statistical analyses (normalized to GAPDH) revealing that spinal nerve ligation (SNL), but not the sham operation (Sham), increased BDNF expression at days 3, 7, 14, and 21 after operation specifically in the ipsilateral (I and IPSI), but not the contralateral (C and CONTRA), dorsal horn. **P < 0.01 vs. Sham IPSI. ^#^P < 0.05, ^##^P < 0.01 vs. SNL day -1. n = 6. IB, Immunoblotting. (**b**) Representative Western blot and statistical analyses (normalized to GAPDH) showing that the SNL-enhanced spinal BDNF expression at day 7 post-operation was attenuated by administering Tet1 mRNA-targeting siRNA (SNL + it + Tet1 RNAi, 3 μg, 10 μL; from day 3–6 after SNL) but not missense siRNA (MS, 3 μg, 10 μL) to animals. **P < 0.01 vs. SNL7D. n = 6. (**c**), Chromatin immunoprecipitation (CHIP) analyses showing that the administration of Tet1 mRNA-targeting siRNA (SNL + it + Tet1 RNAi, 3 μg, 10 μL) attenuated SNL-increased Tet1 binding to the promoter region of the *bdnf* gene. No detectable immunoreactivity was observed in the control IgG-bound precipitates. **P < 0.01 vs. Sham 7D. ^##^P < 0.01 vs. SNL 7D. n = 6. In, input control; CHIP, chromatin immunoprecipitation. (d) Images showed that at day 7 after operation, BDNF immunoreactivity in the ipsilateral dorsal horn was colocalized with NeuN, OX-42, GFAP, and synaptophysin-1. All the BDNF-colocalized as well as GFAP- and OX-42- but rather than NeuN- or synaptophysin-1-positive immunoreactivity were attenuated by daily administration with Tet1 mRNA-targeting siRNA. Scale bar = 50 μm. Thickness = 50 μm.

One study showed that the overexpression of Tet1, which reactivates the methylation-silenced *bdnf* gene, results in increased BDNF expression^5^. Tet1-dependent DNA demethylation is recognized to convert 5 mC to 5 hmC at the CpG sites in the *bdnf* promoter, hence enhancing BDNF expression^10^. Therefore, we next examined whether SNL-induced spinal Tet1-mediated demethylation at the *bdnf* promoter promotes BDNF expression to mediate neuropathic pain. The profile of dorsal horn BDNF expression following response to SNL was first assayed, and then we found that in a manner consistent with SNL-enhanced Tet1 expression, SNL increased the amount of BDNF in the ipsilateral dorsal horn at days 3, 7, 14, and 21 post-operation (Fig. 6a). Daily spinal application of Tet1 mRNA-targeting siRNA (3 μg; 10 μL; day 3–6 post-SNL) reduced SNL-enhanced spinal BDNF expression in the ipsilateral dorsal horn dissected at day 7 after operation (Fig. 6b). Chromatin immunoprecipitation (ChIP) analyses revealed SNL increased the binding of Tet1 to the promoter of the *bdnf* gene, an activity that was reversed by the spinal administration of Tet1 mRNA-targeting siRNA (Fig. 6c; 3 μg; 10 μL). Immunohistochemical images further demonstrated that, at day 7 after SNL, the BDNF immunoreactivity in the dorsal horn coincides with that of neuronal, microglial, astrocytic, and presynaptic vesicle markers. Daily administration with Tet1 mRNA-targeting siRNA attenuated the SNL-enhanced BDNF and all the BDNF-colocalized immunoreactivity (Fig. 6d). Moreover, the immunoreactivity of GFAP and OX-42 but rather than the NeuN or synaptophysin-1 was markedly reduced by focal knockdown of spinal Tet1 expression. These results suggest that Tet1-dependent conversion of 5 mC to 5 hmC at the promoter region in *bdnf* upregulates spinal BDNF expression to participate in the maintenance of neuropathic allodynia.

### Tet1 inhibits SNL-induced DNMT-binding to the *bdnf* promoter

**Figure 7:**
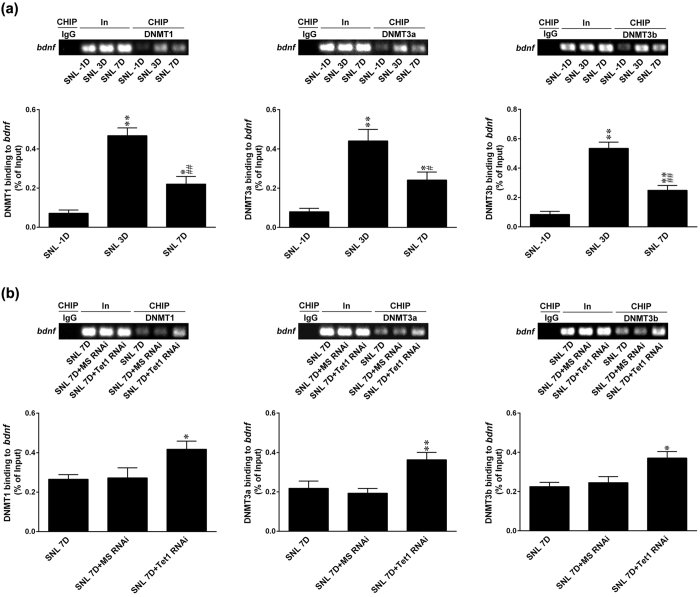
SNL-enhanced Tet1 expression inhibits DNMT binding to the *bdnf* promoter in the ipsilateral dorsal horn. (**a**) SNL-increased DNMT1 (left), DNMT3a (middle), and DNMT3b (right) binding to the *bdnf* promoter at days 3 and 7 post-operation. Notably, the SNL-increased DNMT–*bdnf* promoter binding was partially reversed at day 7 compared with that at day 3 post-operation. *P < 0.05, **P < 0.01 vs. SNL -1D. ^#^P < 0.05, ^##^P < 0.01 vs. SNL 3D. n = 6. No detectable immunoreactivity was observed in the control IgG-bound precipitates. In, input control; CHIP, chromatin immunoprecipitation. (**b**) Tet1 mRNA-targeting siRNA (SNL + it + Tet1 RNAi, 3 μg, 10 μL; from day 3–6 after SNL) enhanced the binding of DNMT1, DNMT3a, and DNMT3b to the promoter region of the *bdnf* gene at day 7 post-SNL. MS, missense siRNA (3 μg, 10 μL). *P < 0.05, **P < 0.01 vs. SNL 7D. n = 6.

In addition to converting 5 mC to 5 hmC at CpG sites, Tet1 itself^14^,^22^ and the newly Tet1-catalysed 5 hmC^14^ both prevent DNA methyltransferase (DNMT) binding to CpG sites, and hence inhibit the conversion of 5C to 5 mC. Based on the above data that the knockdown of spinal Tet1 expression increased 5 mC enrichment at CpG sites in the *bdnf* promoter, we hypothesized that DNMTs and Tet1 act in a compensatory manner in the maintenance of neuropathic pain. Considering DNMT1, DNMT3a and DNMT3b contribute to pain hypersensitivity^16^,^17^, we first investigated the binding of DNMTs to the promoter of the *bdnf* gene in response to neuropathic injury. ChIP analyses indicated that SNL increased the binding of DNMT1, DNMT3a and DNMT3b to the promoter of the *bdnf* gene at day 3 and day 7 after operation (Fig. 7a). Similar to the changes in 5 mC enrichment at the *bdnf* promoter, SNL-enhanced DNMTs binding to the *bdnf* promoter at day 7 was also relatively attenuated and significantly reduced compared with that at day 3 post-SNL. Importantly, focal knockdown of spinal Tet1 expression significantly increased DNMT1, DNMT3a and DNMT3b binding to the *bdnf* promoter at day 7 post SNL (Fig. 7b). Taken together, these results suggest that, in addition to hydroxylating 5 mC to 5 hmC, Tet1 negatively regulates the binding of DNMTs, an activity that was correlated with 5 mC enrichment, at the promoter of the *bdnf* gene to upregulate spinal BDNF expression in the maintenance of nociception hypersensitivity.

## Discussion

**Figure 8:**
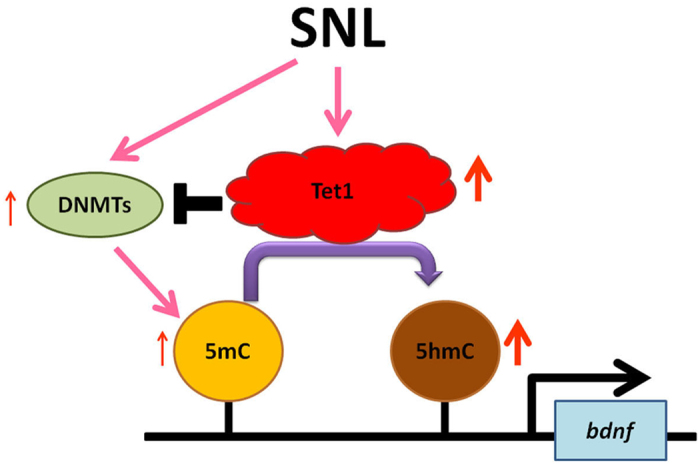
Schematic diagram showing spinal Tet1 contributes to neuropathic pain through DNA demethylation-dependent modification of *bdnf*. SNL facilitates Tet1-dependent demethylation that converts 5 mC to 5 hmC at the promoter of *bdnf*. In addition, the SNL-enhanced Tet1 also inhibits SNL-enhanced DNMTs coupling to 5 mC on *bdnf* promoter. Both these machinery enhance *bdnf* transcription in dorsal horn neurons to underlie neuropathic allodynia development.

In this study, we explored the role of Tet1-mediated DNA demethylation in neuropathic pain development. Our results suggest that spinal Tet1-dependent epigenetic modification of *bdnf* gene transcription mediates the pain hypersensitivity caused by neuropathic injury in rats. Our conclusion is founded on findings that neuropathic injury provoked behavioral allodynia associated with ipsilateral enhanced Tet1 expression in dorsal hron neurons. Focal knockdown of spinal Tet1 expression not only diminished SNL-enhanced spinal Tet1 expression but also reduced the associated allodynia at day 7 after operation. Importantly, detailed analyses revealed that SNL-enhanced spinal BDNF expression at day 7 post SNL is attributed to the coupling of Tet1 to the promoter region of *bdnf* to reactivate *bdnf* transcription by converting 5 mC to 5 hmC, an epigenetic demethylation process. In addition, at the promoter region of the *bdnf* gene, SNL-enhanced DNMT1, DNMT3a, and DNMT3b binding and 5 mC enrichment at day 7 post SNL were significantly reduced at this time point compared with that at day 3 post SNL. Moreover, at day 7 post SNL, Tet1 mRNA-targeting siRNA significantly increased DNMT binding and 5 mC enrichment at the *bdnf* promoter region. Altogether, our results, and given that DNMTs are linked to methylation-dependent epigenetic gene silencing by catalysing 5C to 5 mC^23^,^24^,^25^, suggest that, after neuropathic insults, spinal Tet1 not only prompts demethylation by converting 5 mC to 5 hmC but also inhibits DNMT-dependent methylation to prevent further 5 mC enrichment in the promoter region of *bdnf*. Additionally, synergistically, both of these machineries enhance spinal *bdnf* transcription to underlie neuropathic pain development (Fig. 8). Our findings shed important light on epigenetic markers in neuropathic pain and provide a foundation for understanding the functional role of spinal Tet1-dependent DNA demethylation, which reactivates methylation-silenced genes at specific genomic loci, in the development of pain hypersensitivity. This finding provides a basis for future studies investigating the contribution of DNA demethylation-associated epigenetic modulation of the *bdnf* gene in neuropathic pain and will provide insight towards novel therapeutic strategies for their management.

It is worth noting that a recent study has identified that the Tet1 regulation of Tet2 confers tight regulation of the conversion of 5 mC to 5 hmC^22^. Tet1-deficient animals exhibit the upregulation of genes related to epigenomic modifications and the DNA demethylation pathway, such as Tet2, suggesting that Tet2 might play a compensatory role in the absence of Tet1^15^. In addition, our current study found that nerve injury provoked the Tet1-dependent conversion of 5 mC to 5 hmC in the promoter region of *bdnf*, and then enhanced spinal *bdnf* transcription to underlie neuropathic pain development, all of which was reversed by focal knockdown of spinal Tet1 expression. Similarly, Tet2 was also demonstrated to promote 5 hmC enrichment at the *bdnf* promoter and increase the abundance of BDNF mRNA after ischemic injury; conversely, inhibition of Tet2 reduces the amounts of BDNF mRNA and protein^26^, implying the possible involvement of Tet family members other than Tet1. Particularly, the role of Tet2 in neuropathic pain warrants further study.

BDNF, a member of the neurotrophin family, has been proposed to drive spinal plasticity underlying the central sensitization seen under neuropathic pain conditions^11^,^12^. Studies have demonstrated that drug-induced Tet1 expression upregulates *bdnf* transcription by increasing 5 hmC, an activity that is necessary and sufficient for the demethylation of the *bdnf* promoter^6^,^10^. Consistent with these studies, we observed that SNL-induced spinal Tet1 expression mediates the maintenance of behavioral allodynia by increasing 5 hmC enrichment at the CpG sites of the *bdnf* promoter with subsequent BDNF expression. Nevertheless, a study investigating an animal model of schizophrenia showed prenatal stress-decreased *bdnf* transcription in the frontal cortex and hippocampus was accompanied by the overexpression of Tet1 and increased 5 hmC enrichment at *bdnf* promoter regulatory regions^27^. Although the detailed mechanism is unclear, several potential causes might underlie this discrepancy. First, in contrast to our current study that investigated the DNA demethylation pattern in spinal dorsal horn neurons after SNL, that study focused on the neurons in the frontal cortex and hippocampus after prenatal stress, of which the gene expression profiles might be different from those of the spinal neurons of SNL-induced neuropathic pain rats. In addition, as indicated by Dong and colleagues^27^, there are several regulatory sites in the *bdnf* gene, and whether these regulatory sites play different roles in protein interaction and physiology response, needs clarification in further studies. Moreover, the technique used to quantify DNA demethylation is somewhat different in these studies. Considering that cytosine methylation or hydroxymethylation occurs within the CpG dinucleotide sites, where most transcriptional initiation occurs, crucially modify DNA transcription, we used a specific kit to examine the levels of 5 hmC in the CpGs of the *bdnf* promoter. Our method overcame some of the difficulties in other traditional DNA hydroxymethylation techniques, such as hydroxymethylated DNA Immunoprecipitation, which is used for the detection of any hydroxymethylated cytosine and is not restricted to that in the CpG dinucleotides. Therefore, the differences in techniques may also explain the discrepancy in the results. Nevertheless, the clear-cut mechanism underlying this discrepancy needs further investigations. Notably, at day 7 after operation, SNL increased the dorsal horn BDNF immunoreactivity which coincided with that of neuronal, microglial, astrocytic, and presynaptic vesicle markers. All the BDNF-colocalized immunoreactivity and the GFAP and OX-42, but not the NeuN or synaptophysin immunoreactivity were attenuated by focal knockdown of spinal Tet1 expression. These findings imply that SNL-induced BDNF upregulation resulting from Tet1-associated epigenetic modification might produce downstream activation of neurons, microglia and astrocytes. Nevertheless, it worth notice that in contrast to knockdown of spinal Tet1 expression attenuated BDNF-colocalized as well as the GFAP- and OX-42-labeled immunoreactivity, the SNL-associated spinal Tet1 expression occurred only on neuron but rather than microglia or astrocyte at the identical time point. Though the detail mechanism is unclear, we postulate that neuropathic insult could induce spinal Tet1-dependent epigenetic upregulation of BDNF in neurons that subsequently provoked BDNF expression in the astrocyte and microglia through the neuron-glia interaction, because CNS neurons were demonstrated to regulate microglial activation state and influence basal microglial activities^28^. Yet the detail mechanism needs to be further investigated.

Following peripheral nerve damage, glia cells in the DRG or spinal cord transform from the resting to the activated form and express neurotrophins, such as NGF^29^ and BDNF^30^, to trigger the neural plasticity underlying the pain hypersensitivity through glia-neuron crosstalk^31^. In addition to glial cells, studies have demonstrated neuropathic injury induces BDNF release from primary afferent terminals^32^ and postsynaptic dorsal-horn neurons^32^ to mediate the development of neuropathic pain. Consistent with these studies, our results demonstrated that at day 7 post operation, SNL-induced behavioral allodynia in associated with BDNF expression in the ipsilateral dorsal horn neuron, microglia, and astrocyte. Taken together, these observations suggest that Tet1 activates BDNF expression ipsilaterally in the dorsal horn to underlie SNL-induced behavioral allodynia. Though the data in the current study did not provide information regarding the possible upstream mechanism that trigger the spinal Tet1/BDNF cascade, activation of NMDARs induces BDNF expression^33^; and conversely, spinal injection of an NMDAR antagonist reversed neuropathic-like pain induced by spinal administration of BDNF^34^. We propose that neuropathic injury-facilitated glutamate release is a candidate highly possible for activating Tet1/BDNF cascade to mediate the development of nociceptive hypersensitivity. Nevertheless, further investigations are warranted to explore potential molecules that mediate the SNL-associated spinal Tet1 upregulation.

On the other hand, it is noteworthy that Tet1 is recognized to hyroxylate the 5 mC to 5 hmC and convert 5 hmC to other DNA demethylation intermediates, such as 5-formylcytosine (5fC) and 5-carboxylcytosine (5caC)^3^,^35^,^36^. After deamination, glycosylase-dependent excision, and repair, these DNA demethylation intermediates reverses back to unmodified cytosine for complete active demethylation that initiate transtricption^37^,^38^. In the present study, we linked DNA demethylation to the development of neuropathic pain by assaying abundances of 5 mC and 5 hmC, neverthless, the detail machinery how these demethylation intermediate, such as 5fC, 5caC, and even the 5 hydroxymethylated uracil, contributing to the spinal plasticity underlying pain hypersensitivity requires further study.

Although several members of the DNMT family have been identified to date, only DNMT1, DNMT3a, and DNMT3b possess catalytic activity^39^,^40^,^41^ and are preferentially expressed in neurons pivotally involved in pain hypersensitivity^16^,^17^. Interestingly, recent studies investigating epigenetic modification demonstrated that inflammation and nerve injury impact the global expression of DNMT1, DNMT3a, and DNMT3b in dorsal horn neurons^42^; moreover, following chronic nerve constriction, a model of neuropathic injury, DNMT1, DNMT3a, and DNMT3b are increased in the lumbar spinal cord of rats^17^. Similarly, our present study found that SNL increased the binding of DNMT1, DNMT3a, and DNMT3b to the *bdnf* promoter after operation, implying that SNL increased the spinal DNA methylation of the *bdnf* promoter in rats. Notably, although the binding of DNMT1, DNMT3a, and DNMT3b to the *bdnf* promoter was increased by SNL at days 3 and 7 after operation, we found that they declined at day 7 compared with that at day 3, a result quite different from SNL-enhanced spinal Tet1 expression and 5 hmC enrichment at the *bdnf* promoter that began at day 3 and peaked at day 7 following SNL. Additionally, knockdown of spinal Tet1 expression resulted in further increased DNMT1, DNMT3a, and DNMT3b binding accompanied by increased 5 mC but decreased 5 hmC enrichment at the *bdnf* promoter at day 7. We speculate that, in spinal plasticity, which participates in the development/maintenance of SNL-induced neuropathic pain, Tet1 and DNMT could work together at the *bdnf* genomic locus to mediate active DNA demethylation competitively. Our proposal is supported by recent studies demonstrating that Tet1 prevents DNMT binding to CpG sites^14^,^22^ and that Tet-converted 5 hmC was shown to efficiently inhibit the DNMT binding to the genome loci^14^. In addition, we found that the knockdown of spinal Tet1 expression alleviates SNL-induced allodynia accompanied by increased binding of DNMT1 to the *bdnf* promoter, suggesting that Tet1-mediated *bdnf* demethylation plays a relatively dominant role in the neuropathic pain development compared with DNMTs. Nevertheless, the description of a detail mechanism requires further study.

In contrast, Cheng *et al*. demonstrated that neuropathic injury also provokes allodynia in the contralateral side like a “mirror-image”^29^, Their findings are somewhat different from ours, i.e., SNL-induced allodynia and spinal Tet1 expression occurred specifically in the ipsilateral side. Though the underlying causes for this discrepancy is unclear, the Cheng’s surgical procedure for the neuropathic injury involves holding the suture tight for 10 seconds that is different from ours in which the suture is tight ligated without holding. Whether discrepancy in techniques could induce different neuroplasticity and hence different phenotype of pain hypersensitivity needs further studies to be elucidated.

Beside plastic changes in the spinal cord, cumulative evidences have revealed cortical mechanism^43^, and descending modulation^44^ crucially modify the development/progression of neuropathic pain. In the present study, we demonstrated the contribution of spinal Tet1-dependent plasticity to the development of neuropathic allodynia. The potential impacts of descending controls coming from the higher CNS centers on such a spinal Tet1-associated machinery is highly fascinating and require more experiments to be clarified.

## Methods

### Animal preparations

Male Sprague-Dawley rats (body weight: 200–250 g) were used in this study. All animal procedures were conducted according to the guidelines of the International Association for the Study of Pain^45^. The study protocol was reviewed and approved by the Institutional Review Board of Taipei Medical University.

### Spinal nerve ligation

Spinal nerve ligation rat model was performed as described in our previous studies^46^,^47^. After the animals were anesthetized by isoflurane, the left L5-6 spinal nerves were dissected and tightly ligated with 6-0 silk suture 2–5 mm distal to the dorsal root ganglia after making a middle incision in the skin of the back at the L2-S2 levels. In the sham-operated group, surgical procedures were identical to the nerve ligation group, except the silk suture was placed underneath the left L5-6 spinal nerves but un-ligated.

### Intrathecal catheter

A PE-10 silastic tube was implanted into the intrathecal space around the lumbar enlargement of the rat spinal cord as previous described^48^,^49^, and after implantation of intrathecal catheter, animals were allowed to recover for 3 days.

### Behavioral studies

Tactile sensitivity was evaluated according to the method described by Scha¨fers *et al*.^50^ The calibrated von Frey filaments (0.07–26.0 g) were applied to the plantar surfaces of the hind paws of rats after acclimatization. Mean withdrawal threshold of naive rat was approximately 15 g^46^,^51^. Rats displayed a threshold lower or higher than 15 g were excluded from further study. The withdrawal threshold was assayed one day before surgery to ensure that the animals had normal tactile sensitivity. Motor function was assessed using an accelerating Rotarod apparatus (LE8500, Ugo Basile, Varese, Italy). In short, rats were first subjected to a training session after acclimatization. Over a period of 180 sec, the rod was set to accelerate from 3 to 30 rpm during measuring with a cutoff-time of 180 s. Three measurements were obtained at intervals of 5 min and data were averaged for each test.

### Western blotting

After euthanization, the dorsal quadrants of the lumbar enlargement (L4-5) of animals were dissected and collected for further study. The dissected dorsal horn (L4-5) samples were homogenized in a Tris-HCl solution (25 mM Tris-HCl, 150 mM NaCl, 1% NP-40, 1% sodium deoxycholate, and 0.1% SDS with a complete protease inhibitor mixture) (Roche, Upper Bavaria, Germany). The homogenates were solubilised 1 hour at 4 °C, and centrifugated at 4 °C for 20 min at 18,700 g. A bicinchoninic acid assay (BCA) was used to determine the protein concentration. The proteins were separated on an acrylamide gel and transferred to a polyvinylidene difluoride membrane and then incubated in either rabbit anti-Tet1 (1:500, Millipore, Billerica, Massachusetts), rabbit anti-BDNF (1:4000, Abcam, Cambridge, UK), or mouse anti-Glyceraldehyde 3-phosphate dehydrogenase (GAPDH, 1:4000, Santa Cruz Biotechnology, Santa Cruz, CA) for one hour at room temperature. The blot was then washed and incubated in either peroxidase-conjugated goat anti-rabbit IgG (1:8000, Jackson ImmunoResearch, West Grove, PA) or goat anti-mouse IgG (1:8000, Jackson ImmunoResearch, West Grove, PA) for one hour at room temperature. The protein bands were visualized using an enhanced chemiluminescence detection kit (ECL Plus, Millipore, Billerica, Massachusetts) and then analyzed using a densitometry (Science Lab 2003; Fuji, Tokyo, Japan).

### Immunofluorescence

Under deep isoflurane anesthesia, rats were perfused through the ascending aorta with PBS, followed by 4% paraformaldehyde in PBS (pH 7.4). The harvested spinal cord samples were fixed in the same fixative at 4 °C for 4 hours and then cryoprotected in 30% sucrose solution overnight. For investigating the interactions between Tet1 and neuronal/glial/microglia markers in double-labelling experiments, the spinal sections were incubated at 4 °C overnight with a mixture of rabbit anti-Tet1 (1:200; Millipore, Billerica, Massachusetts) and mouse monoclonal anti-neuronal nuclear antigen (NeuN, a neuronal marker; 1:500; Millipore, Billerica, Massachusetts), mouse anti-glial fibrillary acidic protein (a marker of astroglial cells; 1:1000; Millipore, Billerica, Massachusetts), or mouse anti-integrin αM (OX-42, a marker of microglia; 1:1000; Santa Cruz Biotechnology, Santa Cruz, CA). To examine the distribution of BDNF in the spinal cord of different groups, spinal sections were incubated with rabbit anti-BDNF antibody (1:400, Albcam, Cambridge, UK) and one of the following four antibodies: mouse anti-NeuN (1:500; Millipore, Billerica, Massachusetts), mouse anti-OX-42 (1:1000; Santa Cruz Biotechnology, Santa Cruz, Ca), mouse anti-GFAP (1:1000; Millipore, Billerica, Massachusetts), and mouse anti-synaptophysin-1 (a presynaptic vesicle protein; 1:500; GeneTex, Irvine, Ca). To examine the interactions between Tet1 and 5 hmc in double-labelling experiments, the spinal sections were incubated at 4 °C overnight with a mixture of rabbit anti-Tet1 (1:200; Millipore, Billerica, Massachusetts) and mouse anti-5 hmc (1:200; GeneTex, Irvine, CA). After three times of rinsing with PBS, spinal sections were incubated at 37 °C for one hour with Alexa Fluor 594 conjugated goat anti-rabbit IgG (1:1500; Invitrogen, Grand Island, NY) as well as with Alexa Fluor 488 conjugated goat anti-mouse IgG (1:1500; Invitrogen, Grand Island, NY). Subsequently, coverslips were applied on the spinal cord sections after rinsing. The fluorescent markers were excited when they were detected by a camera-coupled device (X-plorer; Diagnostic Instruments, Inc, USA) using fluorescence microscopy (LEICA DM2500, Germany). For quantity measurement of immunofluorescent intensity, five sections from each spinal cord were selected and seven animals were analyzed in each group. All the parameters used were kept consistent during capturing, and all Images were imported into image J software for analysis.

### Dot blot analysis

The dissected dorsal horn (L4-5) was collected and ground in liquid nitrogen and stored at −80 °C. After extracting the genomic DNA from tissues using a DNA extraction kit (Qiagen, Hilden, Germany), the isolated DNA (100 ng per sample) was denatured in 0.1 M NaOH for 10 min at 95 °C and then neutralized with 1 M NH_4_OAc on ice. Labelled samples were spotted on a positively charged NC membrane at room temperature. The membrane dried naturally at room temperature. After UV cross-linking at 120000 μJ/cm^2^ for 30 sec, the membrane was blocked with 5% milk at room temperature for one hour. The membrane was incubated with the primary antibody—i.e., mouse anti-5 hmC (1:200; GeneTex, Irvine, CA) or mouse anti-5 hmC (1:200; GeneTex, Irvine, CA) at 4 °C overnight after washed. On day 2, the membrane was washed and incubated in goat anti-mouse IgG (1:8000; Jackson ImmunoResearch, West Grove, PA) for one hour at room temperature. An enhanced chemiluminescence detection kit (ECL Plus, Millipore, Billerica, Massachusetts) was used to visualize the blots. Then, the blots were analysed using a densitometry (Science Lab 2003; Fuji, Tokyo, Japan).

### Methylene blue staining

The dot-blot membrane was hybridized with 0.02% methylene blue in 0.3 sodium acetate (pH 5.2) to stain DNA for 10 min. After washing and taking photographs, densitometric analysis of the methylene blue staining was performed with Science Lab 2003 (Fuji, Tokyo, Japan) to validate an equal loading amount of DNA.

### Quantification of 5 hmC/5 mC levels at CpG site of bdnf promoter

The genomic DNA was extracted with a DNA extraction kit (Qiagen, Hilden, Germany). Purification of 5 hmC/5 mC enriched DNA fragments was performed using the Hydroxymethyl Collector Kit (ActiveMotif, Carlsbad, USA) and Methyl Collector Kit (ActiveMotif, Carlsbad, USA), respectively. Following purification, samples were prepared for qPCR using Power SYBR Green (Applied Biosystems, Carlsbad, CA) on an Applied Biosystems^®^ StepOne™ (Applied Biosystems, Carlsbad, CA) under the same conditions described in the gene expression section. Data are presented as % input, which is calculated as 100 × ^2−ΔC*t*^, where ΔCt = the average Ct value of the input triplicate minus the average Ct value of the sample triplicate. The primer for *bdnf* promoter: 5′-GATCCTCCCCTCCTAGCCTAT-3′ and 5′-GAGCCACTAGTTGCCCACAG-3′.

### Chromatin immunoprecipitation

Chromatin immunoprecipitation (ChIP) was performed using commercial kits (Millipore, Billerica, Massachusetts) according to a modified protocol from the manufacturer. The dissected samples were cut into small pieces (1–2 mm^3^) using razor blades. The minced samples were treated with fresh 1% paraformaldehyde in PBS buffer by gentle agitation for 10 minutes at room temperature to cross-link proteins to DNA. Then, the tissues were washed and re-suspended in lysis buffer, and the lysates were sheared by sonication to generate chromatin fragments with an average length of 200–1000 bp. One percent of the sonicated chromatin was saved as an input control for PCR. The chromatin was then immunoprecipitated for two hours at room temperature with antibody against rabbit anti-Tet1 (1:200, Millipore, Billerica, USA), rabbit anti-DNMT1 (1:1000, Abcam, Cambridge, UK), rabbit anti-DNMT3a (1:1000, Abcam, Cambridge, UK), rabbit anti-DNMT3b (1:1000, Abcam, Cambridge, UK) or an equivalent amount of control IgG. The protein-DNA immunocomplexes were precipitated with protein G magnetic beads at 4 °C for overnight. After the beads were washed, they were re-suspended in ChIP elution buffer, incubated with proteinase K at 62 °C for 2 hours, and then incubated at 95 °C for 10 minutes to reverse the protein-DNA cross-links. The purified DNA associated with specific immunoprecipitates or with negative control IgG was isolated and used as a template for polymerase chain reaction (PCR) to amplify the *bdnf* promoter sequences.

### Spinal cord slice preparations

The protocol for tissue preparation was described eleswhere^52^. Briefly, rats were deep anesthetized under isoflurane, and their spinal cords were removed and placed in ice-cold sucrose artificial cerebrospinal fluid (aCSF; sucrose 234 mM, glucose 12 mM, KCl 3.6 mM, MgCl_2_ 1.2 mM, CaCl_2_ 2.5 mM, NaH_2_PO_4_ 1.2 mM, and NaHCO_3_ 25 mM) bubbled with 95% O2/5% CO2. After removing the pia-arachnoid membrane, dissected spinal cords were placed in a shallow groove formed in an agarose block and glued on the stage of a vibratome (DTK1000, Dosaka Co. Ltd., Kyoto, Japan). Transverse spinal slices with a thickness of 400 μm cut at the L4-5 level in the ice-cold sucrose aCSF. The dissected spinal slices were equilibrated in aCSF (NaCl 117 mM, KCl 4.5 mM, CaCl_2_ 2.5 mM, MgCl_2_ 1.2 mM, NaH_2_PO_4_ 1.2 mM, NaHCO_3_ 25 mM, and dextrose 11.4 mM; pH 7.4; bubbled with 95% O2/5% CO2 at room temperature for one hour) before electrophysiological recording. During the recording period, each spinal slice was mounted on a submerged recording chamber and was perfused with oxygenated aCSF at 3–4 ml/min continuously.

### Whole-cell patch-clamp recordings

The lamina II area of the dorsal horn was identified by a translucent band in the superficial dorsal horn on an upright fixed-stage IR-DIC microscope (BX51WI, Olympus, Tokyo, Japan). Spinal lamina I and outer lamina II were selected for recordings, as previously described^53^. Recording pipette (resistance, 5–7 MΩ) was filled with internal solution containing (in mM): 110 Cs^+^ gluconate, 5 TEA, 0.5 CaCl_2_, 5 BAPTA, 10 HEPES, 5 QX314, 5 MgATP, and 0.33 GTP-Tris, pH 7.3, 280 mOsm/L. The recording was discarded if input resistance changed more than 15%. Electrophysiological signals were acquired using the Axon equipment (Molecular Devices, Union City, CA, USA). All signals were sampled by pCLAMP 9.2 via a preamplifier (Axopatch 200B) and an AD-converter (Digidata 1322 A), filtered at 2 kHz, digitized at 10 kHz, and stored for off-line analysis. Miniature EPSCs (mEPSCs) were clamped at −70 mV and recorded in the presence of (−) bicuculline methiodide (10 uM), a GABA_A_ receptor antagonist and tetrodotoxin (1 uM).

### Small-interfering RNA

The duplexes of small-interfering RNAs (siRNAs) targeting to Tet1 were 5′-GGAGGGAUUUCUCACGUUA-3′. The missense nucleotides were 5′-UGAUAUUACCCUGAAUAUG-3′. Either missense or antisense siRNA was daily administered intrathecally for four days with a polyethyleneimine (10 μL, Al 25-kDa, Dharmacon, San Diego, CA)-based gene-delivery system through the implanted catheter (daily for 4 days).

### Data analysis

In this study, all data were analyzed using Prism 6.0 (GraphPad, La Jolla, CA) or SigmaPlot 10.0 (Systat Software, San Jose, CA). Data in the text and figures are expressed as the mean±SEM. We used paired two-tailed Student’s t-test to compare the means between groups. One-way or two-way ANOVAs were used to assess changes in values for serial measurements over time and Tukey’s test was used for post hoc check. Significance was set at p < 0.05.

## Additional Information

**How to cite this article**: Hsieh, M.-C. *et al*. Tet1-dependent epigenetic modification of BDNF expression in dorsal horn neurons mediates neuropathic pain in rats. *Sci. Rep.* **6**, 37411; doi: 10.1038/srep37411 (2016).

**Publisher's note**: Springer Nature remains neutral with regard to jurisdictional claims in published maps and institutional affiliations.

## References

[b1] Denk F. & McMahon S. B. Chronic pain: emerging evidence for the involvement of epigenetics. Neuron 73, 435–444 (2012). 22325197 10.1016/j.neuron.2012.01.012 PMC3996727

[b2] Zhang H. H. . Promoted Interaction of Nuclear Factor-kappaB With Demethylated Purinergic P2X3 Receptor Gene Contributes to Neuropathic Pain in Rats With Diabetes. Diabetes 64, 4272–4284 (2015). 26130762 10.2337/db15-0138

[b3] Ito S. . Role of Tet proteins in 5 mC to 5 hmC conversion, ES-cell self-renewal and inner cell mass specification. Nature 466, 1129–1133 (2010). 20639862 10.1038/nature09303 PMC3491567

[b4] Tahiliani M. . Conversion of 5-methylcytosine to 5-hydroxymethylcytosine in mammalian DNA by MLL partner TET1. Science 324, 930–935 (2009). 19372391 10.1126/science.1170116 PMC2715015

[b5] Kaas G. A. . TET1 controls CNS 5-methylcytosine hydroxylation, active DNA demethylation, gene transcription, and memory formation. Neuron 79, 1086–1093 (2013). 24050399 10.1016/j.neuron.2013.08.032 PMC3816951

[b6] Guo J. U., Su Y., Zhong C., Ming G. L. & Song H. Hydroxylation of 5-methylcytosine by TET1 promotes active DNA demethylation in the adult brain. Cell 145, 423–434 (2011). 21496894 10.1016/j.cell.2011.03.022 PMC3088758

[b7] Rudenko A. . Tet1 is critical for neuronal activity-regulated gene expression and memory extinction. Neuron 79, 1109–1122 (2013). 24050401 10.1016/j.neuron.2013.08.003 PMC4543319

[b8] Zhang R. R. . Tet1 regulates adult hippocampal neurogenesis and cognition. Cell Stem Cell 13, 237–245 (2013). 23770080 10.1016/j.stem.2013.05.006 PMC4474382

[b9] Pan Z. . Hydroxymethylation of microRNA-365-3p Regulates Nociceptive Behaviors via Kcnh2. J Neurosci 36, 2769–2781 (2016). 26937014 10.1523/JNEUROSCI.3474-15.2016 PMC6604871

[b10] Wei Y., Melas P. A., Wegener G., Mathe A. A. & Lavebratt C. Antidepressant-like effect of sodium butyrate is associated with an increase in TET1 and in 5-hydroxymethylation levels in the Bdnf gene. Int. J. Neuropsychopharmacol. 18 (2015). 10.1093/ijnp/pyu032 PMC4368891 25618518

[b11] Miletic G., Hanson E. N. & Miletic V. Brain-derived neurotrophic factor-elicited or sciatic ligation-associated phosphorylation of cyclic AMP response element binding protein in the rat spinal dorsal horn is reduced by block of tyrosine kinase receptors. Neurosci. Lett. 361, 269–271 (2004). 15135945 10.1016/j.neulet.2003.12.029

[b12] Constandil L. . Involvement of spinal cord BDNF in the generation and maintenance of chronic neuropathic pain in rats. Brain Res. Bull. 86, 454–459 (2011). 21864655 10.1016/j.brainresbull.2011.08.008

[b13] Levenson J. M. . Evidence that DNA (cytosine-5) methyltransferase regulates synaptic plasticity in the hippocampus. J. Biol. Chem. 281, 15763–15773 (2006). 16606618 10.1074/jbc.M511767200

[b14] Xu Y. . Genome-wide regulation of 5 hmC, 5 mC, and gene expression by Tet1 hydroxylase in mouse embryonic stem cells. Mol. Cell 42, 451–464 (2011). 21514197 10.1016/j.molcel.2011.04.005 PMC3099128

[b15] Kumar D. . Tet1 Oxidase Regulates Neuronal Gene Transcription, Active DNA Hydroxy-methylation, Object Location Memory, and Threat Recognition Memory. Neuroepigenetics 4, 12–27 (2015). 26644996 10.1016/j.nepig.2015.10.002 PMC4669576

[b16] Pollema-Mays S. L., Centeno M. V., Apkarian A. V. & Martina M. Expression of DNA methyltransferases in adult dorsal root ganglia is cell-type specific and up regulated in a rodent model of neuropathic pain. Front. Cell. Neurosci. 8, 217 (2014). 25152711 10.3389/fncel.2014.00217 PMC4126486

[b17] Wang Y. . Abnormal DNA methylation in the lumbar spinal cord following chronic constriction injury in rats. Neurosci. Lett. 610, 1–5 (2016). 26515497 10.1016/j.neulet.2015.10.048

[b18] Qi F. . Promoter demethylation of cystathionine-beta-synthetase gene contributes to inflammatory pain in rats. Pain 154, 34–45 (2013). 23273102 10.1016/j.pain.2012.07.031

[b19] Williams K. . TET1 and hydroxymethylcytosine in transcription and DNA methylation fidelity. Nature 473, 343–348 (2011). 21490601 10.1038/nature10066 PMC3408592

[b20] Wang W. . Increased expression of sodium channel subunit Nav1.1 in the injured dorsal root ganglion after peripheral nerve injury. Anat Rec (Hoboken) 294, 1406–1411 (2011). 21714116 10.1002/ar.21437 PMC3140573

[b21] Lee C. Y. . Dynamic temporal and spatial regulation of mu opioid receptor expression in primary afferent neurons following spinal nerve injury. Eur. J. Pain 15, 669–675 (2011). 21310637 10.1016/j.ejpain.2010.11.018 PMC3129388

[b22] Freudenberg J. M. . Acute depletion of Tet1-dependent 5-hydroxymethylcytosine levels impairs LIF/Stat3 signaling and results in loss of embryonic stem cell identity. Nucleic Acids Res. 40, 3364–3377 (2012). 22210859 10.1093/nar/gkr1253 PMC3333871

[b23] Sun Y. . DNA Methylation Modulates Nociceptive Sensitization after Incision. PLoS One 10, e0142046 (2015). 26535894 10.1371/journal.pone.0142046 PMC4633178

[b24] Bonasio R., Tu S. & Reinberg D. Molecular signals of epigenetic states. Science 330, 612–616 (2010). 21030644 10.1126/science.1191078 PMC3772643

[b25] Feng S., Jacobsen S. E. & Reik W. Epigenetic reprogramming in plant and animal development. Science 330, 622–627 (2010). 21030646 10.1126/science.1190614 PMC2989926

[b26] Miao Z. . Altering 5-hydroxymethylcytosine modification impacts ischemic brain injury. Hum. Mol. Genet. 24, 5855–5866 (2015). 26231219 10.1093/hmg/ddv307 PMC4581609

[b27] Dong E. . Brain-derived neurotrophic factor epigenetic modifications associated with schizophrenia-like phenotype induced by prenatal stress in mice. Biol. Psychiatry 77, 589–596 (2015). 25444166 10.1016/j.biopsych.2014.08.012 PMC4333020

[b28] Eyo U. B. & Wu L. J. Bidirectional microglia-neuron communication in the healthy brain. Neural Plast. 2013, 456857 (2013). 24078884 10.1155/2013/456857 PMC3775394

[b29] Cheng C. F. . Mirror-image pain is mediated by nerve growth factor produced from tumor necrosis factor alpha-activated satellite glia after peripheral nerve injury. Pain 155, 906–920 (2014). 24447514 10.1016/j.pain.2014.01.010

[b30] Coull J. A. . BDNF from microglia causes the shift in neuronal anion gradient underlying neuropathic pain. Nature 438, 1017–1021 (2005). 16355225 10.1038/nature04223

[b31] Zhuo M., Wu G. & Wu L. J. Neuronal and microglial mechanisms of neuropathic pain. Mol. Brain 4, 31 (2011). 21801430 10.1186/1756-6606-4-31 PMC3163530

[b32] Fawcett J. P. . Evidence that brain-derived neurotrophic factor from presynaptic nerve terminals regulates the phenotype of calbindin-containing neurons in the lateral septum. J Neurosci 20, 274–282 (2000). 10627605 10.1523/JNEUROSCI.20-01-00274.2000 PMC6774122

[b33] Song Q. X., Chermansky C. J., Birder L. A., Li L. & Damaser M. S. Brain-derived neurotrophic factor in urinary continence and incontinence. Nat Rev Urol 11, 579–588 (2014). 25224451 10.1038/nrurol.2014.244 PMC6946056

[b34] M’Dahoma S. . Respective pharmacological features of neuropathic-like pain evoked by intrathecal BDNF versus sciatic nerve ligation in rats. Eur. Neuropsychopharmacol. 25, 2118–2130 (2015). 26343858 10.1016/j.euroneuro.2015.07.026

[b35] Ito S. . Tet proteins can convert 5-methylcytosine to 5-formylcytosine and 5-carboxylcytosine. Science 333, 1300–1303 (2011). 21778364 10.1126/science.1210597 PMC3495246

[b36] Bhutani N., Burns D. M. & Blau H. M. DNA demethylation dynamics. Cell 146, 866–872 (2011). 21925312 10.1016/j.cell.2011.08.042 PMC3236603

[b37] Branco M. R., Ficz G. & Reik W. Uncovering the role of 5-hydroxymethylcytosine in the epigenome. Nat Rev Genet 13, 7–13 (2012). 10.1038/nrg3080 22083101

[b38] Okano M., Xie S. & Li E. Cloning and characterization of a family of novel mammalian DNA (cytosine-5) methyltransferases. Nat. Genet. 19, 219–220 (1998). 9662389 10.1038/890

[b39] Chedin F. The DNMT3 family of mammalian de novo DNA methyltransferases. Prog. Mol. Biol. Transl. Sci. 101, 255–285 (2011). 21507354 10.1016/B978-0-12-387685-0.00007-X

[b40] Qin W., Leonhardt H. & Pichler G. Regulation of DNA methyltransferase 1 by interactions and modifications. Nucleus (Calcutta) 2, 392–402 (2011). 10.4161/nucl.2.5.17928 21989236

[b41] Tochiki K. K., Cunningham J., Hunt S. P. & Geranton S. M. The expression of spinal methyl-CpG-binding protein 2, DNA methyltransferases and histone deacetylases is modulated in persistent pain states. Mol. Pain 8, 14 (2012). 22369085 10.1186/1744-8069-8-14 PMC3351747

[b42] He Y. F. . Tet-mediated formation of 5-carboxylcytosine and its excision by TDG in mammalian DNA. Science 333, 1303–1307 (2011). 21817016 10.1126/science.1210944 PMC3462231

[b43] Zhuo M. Neural Mechanisms Underlying Anxiety-Chronic Pain Interactions. Trends Neurosci. 39, 136–145 (2016). 26878750 10.1016/j.tins.2016.01.006

[b44] Porreca F., Ossipov M. H. & Gebhart G. F. Chronic pain and medullary descending facilitation. Trends Neurosci. 25, 319–325 (2002). 12086751 10.1016/s0166-2236(02)02157-4

[b45] Zimmermann M. Ethical guidelines for investigations of experimental pain in conscious animals. Pain 16, 109–110 (1983). 6877845 10.1016/0304-3959(83)90201-4

[b46] Lin T. B. . Modulation of Nerve Injury-induced HDAC4 Cytoplasmic Retention Contributes to Neuropathic Pain in Rats. Anesthesiology 123, 199–212 (2015). 25871743 10.1097/ALN.0000000000000663

[b47] Lin T. B. . Neuropathic Allodynia Involves Spinal Neurexin-1beta-dependent Neuroligin-1/Postsynaptic Density-95/NR2B Cascade in Rats. Anesthesiology 123, 909–926 (2015). 26263430 10.1097/ALN.0000000000000809

[b48] Lin T. B. . Fbxo3-Dependent Fbxl2 Ubiquitination Mediates Neuropathic Allodynia through the TRAF2/TNIK/GluR1 Cascade. J Neurosci 35, 16545–16560 (2015). 26674878 10.1523/JNEUROSCI.2301-15.2015 PMC6605509

[b49] Lin T. B. . VPS26A-SNX27 Interaction-Dependent mGluR5 Recycling in Dorsal Horn Neurons Mediates Neuropathic Pain in Rats. J Neurosci 35, 14943–14955 (2015). 26538661 10.1523/JNEUROSCI.2587-15.2015 PMC6605230

[b50] Schafers M., Svensson C. I., Sommer C. & Sorkin L. S. Tumor necrosis factor-alpha induces mechanical allodynia after spinal nerve ligation by activation of p38 MAPK in primary sensory neurons. J Neurosci 23, 2517–2521 (2003). 12684435 10.1523/JNEUROSCI.23-07-02517.2003 PMC6742090

[b51] Chung J. M., Kim H. K. & Chung K. Segmental spinal nerve ligation model of neuropathic pain. Methods Mol. Med. 99, 35–45 (2004). 15131327 10.1385/1-59259-770-X:035

[b52] Lai C. Y. . Spinal Fbxo3-Dependent Fbxl2 Ubiquitination of Active Zone Protein RIM1alpha Mediates Neuropathic Allodynia through CaV2.2 Activation. J Neurosci 36, 9722–9738 (2016). 27629721 10.1523/JNEUROSCI.1732-16.2016 PMC6601949

[b53] Zhou H. Y., Chen S. R., Chen H. & Pan H. L. Opioid-induced long-term potentiation in the spinal cord is a presynaptic event. J Neurosci 30, 4460–4466 (2010). 20335482 10.1523/JNEUROSCI.5857-09.2010 PMC2852319

